# Complete genome sequences of *Castellaniella ginsengisoli* associated with mortality events in avian species

**DOI:** 10.1128/mra.00703-24

**Published:** 2025-01-28

**Authors:** Yi-Chen Luo, Jenny Nicholds, Tatum D. Mortimer, Grazieli Maboni

**Affiliations:** 1 Department of Infectious Diseases, Athens Veterinary Diagnostic Laboratory, The University of Georgia, Athens, Georgia, USA; 2 Poultry Diagnostic and Research Center, Department of Population Health, The University of Georgia, Athens, Georgia, USA; University of Maryland School of Medicine, Baltimore, Maryland, USA

**Keywords:** whole-genome sequencing, long-read-first hybrid assembly, avian species, chicken, *Castellaniella* spp., *Castellaniella ginsengisoli*

## Abstract

*Castellaniella ginsengisoli* is a potential bacterial pathogen that affects chickens. We present 22 complete genome sequences of clinical isolates to facilitate the genomic analysis and the development of diagnostic tools.

## ANNOUNCEMENT

The genus *Castellaniella* was first proposed under the family *Alcaligenaceae* in 2006 with its unique phenotypic and chemotaxonomic characterization (1). *Castellaniella* spp. is typically found in soil and water ecosystems and contributes to the breakdown of pollutants in wastewater and denitrification in poultry manure composting (2–5). Previously, it was unknown to cause disease in mammals until scattered reports unveiled its potential involvement in animal health issues (6, 7). Of particular concern is the emergence of *Castellaniella ginsengisoli* in broiler breeders of the Southeastern United States. Instances of lameness, cellulitis, and mortality events have been linked to their presence, resulting in substantial economic losses within the poultry industry (8). It is critical to understand the genomic features, virulence, and antimicrobial resistance mechanisms of *Castellaniella ginsengisoli* for effective disease management. The availability of whole-genome sequences of *Castellaniella ginsengisoli* isolated from avian species would allow researchers to explore such genomic features in clinical isolates. Furthermore, it can potentially enrich the *Castellaniella ginsengisoli* genome database, thereby facilitating the pinpointing of candidate genome regions for developing diagnostic assays.

A total of 22 *Castellaniella ginsengisoli* isolates were obtained from chickens presenting with clinical signs. Swabs were taken from affected lesions for aerobic culture on 5% sheep blood agar for 48 hour incubation. Sanger sequencing of the 16S rRNA (9) gene initially identified these isolates as *Castellaniella* spp. Subsequently, isolates were stored in either Tryptic Soy Broth with 10% glycerol or Brain Heart infusion broth with 15% glycerol. DNA extraction was performed using the GenElute Bacterial Genomic DNA Extraction Kit (Sigma-Aldrich). All isolates were sequenced by both long-read sequencing and short-read sequencing. Oxford Nanopore Technology (ONT) libraries were prepared using the SQK-RBK114-24 rapid barcoding kit and sequenced on R.10.4.1 flowcells (ONT, United Kingdom) with MinION. The Illumina DNA prep kit (Illumina, San Diego, CA) was used for library preparations, and MiSeq Reagent Kit v3 (Illumina, San Diego, CA) was used for MiSeq sequencing synthesizing maximum paired-end read length of 300 bp. For read processing, default parameters were used unless indicated otherwise. Nanopore reads were base called using Dorado v0.4.3 (10) with sup model (dna_r10.4.1_e8.2_400bps_sup@v4.2.0) and with q-score filtration (--min-qscore 9), and Porechop v0.2.4 (11) was used for adapter trimming. Paired-end Illumina reads were quality trimmed using BBtools v39.00 (12). We used Trycycler v0.5.4(13) to generate consensus by combining long-read-only assemblies from multiple long-read assemblers (Miniasm/Minipolish v0.1.3 [14], Raven v1.8.1 [15], and Flye v2.9.2 [16]). Trycycler v0.5.4 (13) circularized contig sequences generated from different assemblers by aligning the start and end of one contig to another to determine whether there is a need to add or remove any sequences from a contig for circularization, but did not perform contig rotation due to the absence of the default starting sequence in the genomes. Circularized contigs were polished with long-read polisher Medaka v1.11.3 (17) and short-read polishers Polypolish v0.6.0 (18) and POLCA (19). The long-read N50 was assessed with Nanostat v.1.6.0 (20), while the assembly completeness was assessed using BUSCO v.5.5.0 (21).

All samples yielded a single, circularized contig with a genome size of approximately 3 million bp, a minimum coverage depth of 66×, and a GC content of 66%. BUSCO identified over 97% of single-copy genes as complete, indicating the completeness of assemblies. Relevant statistics are shown in Table 1. The authors deemed the current study retrospective, so no ethical committee was engaged in reviewing this study.

**TABLE 1:** Metrics and accession numbers of genome sequences of *Castellaniella ginsengisoli* isolated from chickens

Sample ID	Illumina		Nanopore - Trycycler			Genome size (bp)	Accession numbers		
	Reads #	Coverage (×)	Reads #	Raw-read N50^*a*^	Coverage (×)^*b*^		ONT	Illumina	Assembly
148131	215,397	20.2	117,683	12,986	275	2,958,440	SRR29307032	SRR29152617	CP158258
145852	122,138	11	94,273	14,575	240	2,939,522	SRR29307033	SRR29152616	CP158259
145849	175,154	17	40,650	14,449	102	2,939,577	SRR29307035	SRR29152605	CP158261
150221	237,638	23	134,977	12,547	307	2,937,616	SRR29307031	SRR29152602	CP158257
144863	206,071	19	158,200	11,386	313	3,021,650	SRR29307036	SRR29152601	CP158262
143936	3,143,096	251	45,273	15,942	111	2,911,400	SRR29307037	SRR29152600	CP158263
143769	2,982,091	236	84,763	14,336	203	2,958,439	SRR29307019	SRR29152599	CP158265
141555	258,256	25	40,466	12,782	90	2,951,780	SRR29307021	SRR29152598	CP158267
130416	250,770	24	56,916	12,501	126	2,948,848	SRR29307023	SRR29152597	CP158269
124566	355,082	34	64,249	10,573	116	2,948,846	SRR29307038	SRR29152596	CP158272
124953	3,690,024	293	43,600	9,582	65.3	2,948,849	SRR29307027	SRR29152615	CP158271
130308	238,474	22	49,830	13,146	112	2,948,845	SRR29307024	SRR29152614	CP158270
150964	409,243	39	99,248	11,220	183	2,931,857	SRR29307030	SRR29152613	CP158256
143811	199,500	20	46,084	18,155	148	2,880,193	SRR29307018	SRR29152612	CP158264
143751	177,014	17	52,758	11,920	106.3	2,880,196	SRR29307020	SRR29152611	CP158266
124370	3,116,278	244	60,880	9,714	86	2,948,785	SRR29307039	SRR29152610	CP158273
145850	5,441,192	403	70,746	14,389	168	2,939,560	SRR29307034	SRR29152609	CP158260
140124	2,464,502	25	40,173	9,735	66	2,937,069	SRR29307022	SRR29152608	CP158268
151108	2,675,533	213	147,388	9,696	267	2,948,848	SRR29307029	SRR29152607	CP158255
151836	235,624	22	103,002	10,409	191	3,032,368	SRR29307028	SRR29152606	CP158254
153271	288,774	28	137,980	10,717	262	2,939,711	SRR29307026	SRR29152604	CP158253
153920	1,512,701	119	65,243	12,182	130	3,014,323	SRR29307025	SRR29152603	CP158252

^*a*^ The N50 of raw reads is assessed using Nanostat v.1.6.0.

^*b*^ Coverage (×) is calculated in Trycycler v0.5.4 using Nanopore long read as input.

## Data Availability

The data were deposited to the NCBI GenBank database under the BioProject accession no. PRJNA1115805. The complete sequences and their corresponding raw reads have been deposited in the GenBank and the Sequence Read Archive (SRA) (Table 1).
